# Aberrantly Expressed Galectin-9 Is Involved in the Immunopathogenesis of Anti-MDA5-Positive Dermatomyositis-Associated Interstitial Lung Disease

**DOI:** 10.3389/fcell.2021.628128

**Published:** 2021-03-25

**Authors:** Lin Liang, Ya-Mei Zhang, Ya-Wen Shen, Ai-Ping Song, Wen-Li Li, Li-Fang Ye, Xin Lu, Guo-Chun Wang, Qing-Lin Peng

**Affiliations:** ^1^Department of Rheumatology, Beijing Key Lab for Immune-Mediated Inflammatory Diseases, China–Japan Friendship Hospital, Beijing, China; ^2^Graduate School of Peking Union Medical College, Beijing, China; ^3^Department of Pathology, China–Japan Friendship Hospital, Beijing, China

**Keywords:** galectin-9, interstitial lung disease, melanoma differentiation-associated gene 5, biomarker, dermatomyositis

## Abstract

**Background:**

Dermatomyositis (DM) associated rapidly progressive interstitial lung disease (RP-ILD) has high mortality rate and poor prognosis. Galectin-9 (Gal-9) plays multiple functions in immune regulation. We investigated Gal-9 expression in DM patients and its association with DM-ILD.

**Methods:**

A total of 154 idiopathic inflammatory myopathy patients and 30 healthy controls were enrolled in the study. Cross-sectional and longitudinal studies were used to analyze the association between serum Gal-9 levels and clinical features. Enzyme-linked immunosorbent assay and qRT-PCR were used to examine Gal-9 expression in the sera and isolated peripheral blood mononuclear cells (PBMCs) from DM patients. Immunohistochemistry was performed to analyze the expression of Gal-9 and its ligand (T-cell immunoglobulin mucin (Tim)-3 and CD44) in lung tissues from anti-melanoma differentiation-associated gene 5 (MDA5)-positive patients. The effect of Gal-9 on human lung fibroblasts (MRC-5) was investigated *in vitro*.

**Results:**

Serum Gal-9 levels were significantly higher in DM patients than in immune-mediated necrotizing myopathy patients and healthy controls (all *p* < 0.001). Higher serum Gal-9 levels were observed in anti-MDA5-positive DM patients than in anti-MDA5-negative DM patients [33.8 (21.9–44.7) vs. 16.2 (10.0–26.9) ng/mL, *p* < 0.001]. Among the anti-MDA5-positive DM patients, serum Gal-9 levels were associated with RP-ILD severity. Serum Gal-9 levels were significantly correlated with disease activity in anti-MDA5-positive DM patients in both cross-sectional and longitudinal studies. PBMCs isolated from anti-MDA5-positive DM patients (3.7 ± 2.3 ng/mL) produced higher levels of Gal-9 than those from immune-mediated necrotizing myopathy patients (1.1 ± 0.3 ng/mL, *p* = 0.022) and healthy controls (1.4 ± 1.2 ng/mL, *p* = 0.045). The mRNA levels of Gal-9 were positively correlated with the levels of type-I interferon-inducible genes MX1 (*r* = 0.659, *p* = 0.020) and IFIH1 (*r* = 0.787, *p* = 0.002) in PBMCs from anti-MDA5-positive DM patients. Immunohistochemistry revealed increased Gal-9 and Tim-3 expression in the lung tissues of patients with DM and RP-ILD. *In vitro* stimulation with Gal-9 protein increased CCL2 mRNA expression in MRC-5 fibroblasts.

**Conclusions:**

Among anti-MDA5-positive DM patients, Gal-9 could be a promising biomarker for monitoring disease activity, particularly for RP-ILD severity. Aberrant expression of the Gal-9/Tim-3 axis may be involved in the immunopathogenesis of DM-ILD.

## Introduction

Dermatomyositis (DM) is a subgroup of idiopathic inflammatory myopathies (IIMs) which frequently involves in muscle, skin, lung and other organs (Selva-O’Callaghan et al., 2018). Myositis-specific autoantibodies (MSAs) are common in patients with DM and can help define the disease into more homogeneous subgroups (Li et al., 2019). Patients carrying anti-melanoma differentiation-associated gene 5 (MDA5) antibodies are likely to develop interstitial lung disease (ILD), particularly rapidly progressive (RP)-ILD, which has a high mortality rate and poor prognosis (Johnson et al., 2016; Abe et al., 2017; Moghadam-Kia et al., 2017). Previous studies suggested that activation of monocytes, macrophages, neutrophils, and CD4 + T helper (Th) 1 cells, and increased expression of CD4+CXCR4+ T cells and interferon (IFN)-γ contribute to the development of DM-ILD (Ishikawa et al., 2018; Wang et al., 2019b; Matsuda et al., 2020; Zuo et al., 2020). However, the pathogenesis of DM-ILD remains to be fully understood.

Galectins are a family of proteins that bind to β-galactoside-containing glycans (Thiemann and Baum, 2016). In this family, galectin-9 (Gal-9) has been detected in monocytes, macrophages, endothelial cells, fibroblasts, and Kupffer cells (Golden-Mason and Rosen, 2017; Wiersma et al., 2013). Gal-9 plays multiple functions in immune regulation inducing cell migration, activation, and apoptosis (Bacigalupo et al., 2013; Fujita et al., 2017). Several ligands of Gal-9, including T-cell immunoglobulin mucin (Tim)-3, CD44, and protein disulfide isomerase have been identified (Zhu et al., 2005; Bi et al., 2011; Wu et al., 2014). Th1 cells, Th17 cells, and alveolar macrophages were reported to express Tim-3 (Zhu et al., 2011; Jiao et al., 2016; Wang et al., 2019a). Lu and colleagues (Lu et al., 2015a) demonstrated that the Gal-9/Tim-3 pathway suppressed the respiratory syncytial virus-induced lung inflammation by inhibiting the Th1 and Th17 immune response in mice. Additionally, it has been reported that the CD44-dependent interaction with hyaluronan was inhibited by Gal-9 on human lung fibroblast cells, thus protecting against lung fibrosis in patients with cryptogenic organizing pneumonia (Katoh et al., 2015). A growing body of reports have suggested the importance of Gal-9 in various diseases such as cancer and autoimmune diseases. It has been demonstrated that Gal-9 is correlated with disease activity and strongly correlated with IFN score in patients with systemic lupus erythematosus (van den Hoogen et al., 2018). Furthermore, Wiersma et al. (2019) reported that Gal-9 activates peptidyl arginine deiminase 4 in granulocytes and promotes immunopathology in patients with rheumatoid arthritis.

Increased serum Gal-9 levels were reported in juvenile DM and were demonstrated to correlate with disease activity (Bellutti Enders et al., 2014; Wienke et al., 2019). However, very limited data is available in the relation between the serum levels of Gal-9 and MSA types or DM-ILD. In addition, the effect of Gal-9 has not been established in the pathogenesis of DM-ILD. Therefore, in this study, we systemically investigated expression of Gal-9 in patients with DM, and its impact on the development of DM-ILD.

## Materials and Methods

### Patient Sampling

A total of 154 patients with IIM including 129 patients with DM and 25 patients with immune-mediated necrotizing myopathy (IMNM) from China–Japan Friendship Hospital were enrolled in this study. DM was diagnosed based on the 2017 ACR/EULAR IIM criteria (Lundberg et al., 2017), and IMNM was diagnosed using 2004 ENMC IMNM criteria (Hoogendijk et al., 2004). Patients younger than 16 years of age and those exhibiting complications or other connective tissue diseases (such as rheumatoid arthritis, systemic lupus erythematosus and systemic sclerosis) were excluded from the study to exclude the potential bias resulted from high Gal-9 levels caused by other diseases. Additionally, we enrolled 30 healthy, age- and sex-matched volunteers as healthy controls (HCs). Informed consent was obtained from all participants. The Ethical Review Committee of the China–Japan Friendship Hospital (2019-25-K19) approved this study.

We collected the demographic features, clinical features, and laboratory data of the patients from electronic medical records. In the longitudinal study, 21 anti-MDA5-positive patients with DM were followed up for 1–42 months. The median follow-up duration was 22 months. We collected the blood samples during the follow-up period at each hospitalization. The interval between the two sample collections from a single patient was 1–25 months. ILD was confirmed by computed tomography and pulmonary function analyses (Travis et al., 2013). According to the American Thoracic Society and the European Respiratory Society, RP-ILD was diagnosed by a worsening radiologic interstitial status and the presence of respiratory symptoms such as progressive dyspnea and hypoxemia within 3 months from the onset of respiratory issues (Gono et al., 2010; Travis et al., 2013). For pulmonary function examination, the results of forced vital capacity (FVC), forced expiratory volume in 1 s (FEV1) and diffusing capacity of carbon monoxide (DLco) were collected. The myositis disease activity was assessed by 10-cm visual analog scales (VAS) for muscle, six extramuscular organ systems including constitutional (fever, involuntary weight loss and fatigue), cutaneous (skin), joint, gastrointestinal, pulmonary, and cardiac, and the physician’s global assessment (PGA).

### Detection of Serum Gal-9 and MSA

An enzyme-linked immunosorbent assay (ELISA) kit (R&D Systems, Minneapolis, MN, United States) was used to measure the serum Gal-9 levels. Additionally, MSAs were detected using an immunoblot assay kit (Euroimmun, Lübeck, Germany). Anti-3-hydroxy-3-methyl coenzyme A reductase protein autoantibodies were measured by ELISA (Inova Diagnostics Inc., San Diego, CA, United States). These assays were performed according to the manufacturer’s instructions.

### Cell Culture and Treatment

#### Peripheral Blood Mononuclear Cells (PBMCs) Culture and Treatment

PBMCs were isolated by centrifugation on a Histopaque density gradient (Sigma-Aldrich, St. Louis, MO, United States). The isolated PBMCs were seeded into 96-well plates at 5 × 10^5^ cells/mL in Roswell Park Memorial Institute 1640 medium (Gibco, Grand Island, NY, United States) supplemented with 10% fetal bovine serum (Gibco) and 100 U/μg/mL penicillin/streptomycin (Gibco) at 37°C and 5% CO2 for 48 h. The supernatant was collected by centrifugation. An ELISA kit was used to determine Gal-9 levels in the supernatant, as described.

#### MRC-5 Fibroblasts Culture and Treatment

MRC-5 human lung fibroblasts were obtained from American Type Culture Collection (Manassas, VA, United States) and seeded into 6-well plates at 1 × 10^5^ cells/mL in minimum essential medium (Hyclone, Logan, UT, United States) containing 10% fetal bovine serum (Gibco), 1% non-essential amino acids (Gibco), and 100 U/μg/mL penicillin/streptomycin (Gibco) at 37°C and 5% CO2 for 8 h. Next, Gal-9 (Biolegend, San Diego, CA, United States) or transforming growth factor-β (TGF-β) (Peprotech, Rocky Hill, NJ, United States) were added. The proliferation of MRC-5 fibroblasts stimulated with Gal-9 for 24 or 48 h was tested with a luminescent cell viability assay kit (Promega Corporation, Madison, WI, United States) according to the manufacturer’s instructions.

### Quantitative Reverse-Transcription Polymerase Chain Reaction (qRT-PCR)

Total RNA was isolated from PBMCs or cultured MRC-5 fibroblasts stimulated with Gal-9 for 24 h using TRIzol reagent (Invitrogen, Carlsbad, CA, United States). The mRNA levels were tested by SYBR-Green-based qRT-PCR. Glyceraldehyde-3-phosphate dehydrogenase (GAPDH) was used as an internal control. Primers of target genes [Gal-9, myxovirus resistance 1 (MX1), interferon induced with helicase C domain 1 (IFIH1), monocyte chemoattractant protein-1(CCL2), interleukin 1β (IL-1β), IL-2, IL-4, IL-6, IL-8, IL-10, IL-17A, tumor necrosis factor-α (TNF-α), IFNγ, CCL18, C-X-C motif chemokine ligand 4 (CXCL4), and CXCL10] are shown in Supplementary Table 1. The 2^–Δ^ ^*Ct*^ method was used to calculate the relative gene levels.

### Immunohistochemistry

The lung tissue samples were obtained by surgical resection or percutaneous lung biopsy. Tissues were fixed in 10% formalin and embedded in paraffin, and subjected to antigen retrieval by heating and treatment with 3% hydrogen peroxide for 15 min. After incubation with rabbit anti-Gal-9 monoclonal antibody (1:500 dilution; Abcam, Cambridge, United Kingdom), anti-Tim-3 (1:400 dilution; Proteintech, Rocky Hill, NJ, United States), and anti-CD44 (1:50 dilution; Biolegend) overnight at 4°C, goat anti-rabbit IgG antibody (Gene Tech Shanghai Company Limited, Shanghai, China) was incubated with the tissue sections for 30 min at room temperature. 3,3’-Diaminobenzidine (Gene Tech Shanghai Company Limited) was used as a chromogenic reagent and hematoxylin was used for counterstaining.

### Western Blot Analysis

MRC-5 fibroblasts were stimulated with Gal-9 or TGF-β for 48 h. Total protein from the cells was extracted by adding protein lysis buffer to the cells. Western blotting was conducted using primary antibodies of rabbit polyclonal anti-smooth muscle actin (SMA) (1:1000 dilution; Proteintech) and mouse monoclonal anti-GAPDH (1:1000 dilution; Abcam), followed by secondary antibodies including peroxidase-conjugated goat anti-mouse IgG (1:5000 dilution; Abcam) and peroxidase-conjugated goat anti-rabbit IgG (1:5000 dilution; Abcam). Enhanced chemiluminescence substrate (Thermo Fisher Scientific, Waltham, MA, United States) was added to the membranes. Quantitative protein densitometry was performed with ImageJ software (NIH, Bethesda, MD, United States).

### Statistical Analysis

Data analysis was performed using GraphPad Prism V.7.01 (GraphPad, Inc., San Diego, CA, United States) and SPSS Version 22 (SPSS, Inc., Chicago, IL, United States). Numbers (percentages), mean ± standard deviation, or median values and interquartile range (IQR) were used to express the data. For two-group comparisons, Student’s t-test or Mann–Whitney U-test was used for continuous variables, and the chi-squared test was used for categorical variables. For comparison among multiple groups, the Kruskal–Wallis H-test was performed. The correlations of normally and non-normally distributed data were measured using Pearson’s correlation and Spearman’s correlation, respectively. Longitudinal data were analyzed with the generalized estimating equation model. *p*-values below 0.05 were considered to indicate statistical significance.

## Results

### Patient Characteristics

Patients with IIM (*n* = 154) were enrolled in the present study. The demographics, clinical manifestations, and laboratory characteristics of patients with DM and IMNM are shown in Table 1. Furthermore, DM patients were divided into the anti-MDA5-positive group and anti-MDA5-negative group. The baseline characteristics between the two groups were shown in Supplementary Table 2. All patients received corticosteroids during hospitalization. The corticosteroid dosages were between 0.5–1 mg/kg. Approximately 84.4% of patients (130 of 154) were administered corticosteroids and immunosuppressive agents (methotrexate, cyclophosphamide, ribavirin, intravenous immunoglobulin, hydroxychloroquine, and mycophenolate mofetil).

**TABLE 1:** Baseline characteristics observed in DM patients and IMNM patients.

Characteristics	DM patients	IMNM patients	*p*
	(*n* = 129)	(*n* = 25)	
Gender, no. (%)			0.206
Female	89 (69.0%)	14 (56.0%)	−
Male	40 (31.0%)	11 (44.0%)	−
Age of onset, median (IQR), years	49.0 (39.0–57.0)	47.0 (31.5–57.5)	0.459
Disease duration, median (IQR), months	6 (2–24)	10 (5–24)	0.282
Clinical features, no. (%)			
ILD	89 (69.0%)	0 (0%)	< 0.001
RP-ILD	26 (20.2%)	0 (0%)	0.008
Muscle weakness	55 (42.6%)	20 (80.0%)	0.001
Myalgia	36 (27.9%)	6 (24.0%)	0.688
Mechanic’s hands	37 (28.7%)	0 (0%)	0.001
Raynaud’s phenomenon	10 (7.8%)	0 (0%)	0.368
Heliotrope rash	95 (73.6%)	3 (12.0%)	< 0.001
Gottron’s papules	69 (53.5%)	1 (4.0%)	< 0.001
Skin ulceration	25 (19.4%)	0 (0%)	0.015
Calcinosis	5 (3.9%)	0 (0%)	1.000
Arthritis/arthralgia	35 (27.1%)	1 (4.0%)	0.010
Dysphagia	18 (14.0%)	3 (12.0%)	1.000
Malignancy	1 (0.8%)	0 (0%)	1.000
Pulmonary function test, median (IQR)			
FVC%	84.9 (74.1–104.1)^a^	77.0 (62.8–88.7)^g^	0.133
FEV_1_%	79.9 (76.0–83.6)^a^	80.1 (73.6–83.9)^g^	0.787
DLco%	63.1 (50.8–77.9)^b^	74.3 (68.9–91.1)^h^	0.017
Laboratory features			
MSA, no. (%)	116 (89.9%)	17 (68.0%)	0.003
Anti-MDA5-positive	56 (43.4%)	0 (0%)	−
Anti-ARS-positive	24 (18.6%)	0 (0%)	−
Anti-TIF1-γ-positive	12 (9.3%)	0 (0%)	−
Anti-NXP-2-positive	11 (8.5%)	0 (0%)	−
Anti-Mi-2-positive	7 (5.4%)	0 (0%)	−
Anti-SAE-positive	6 (4.7%)	0 (0%)	−
Anti-SRP-positive	0 (0%)	10 (40.0%)	−
Anti-HMGCR-positive	0 (0%)	7 (28.0%)	−
CK (IU/L), median (IQR)	78 (36–214)^c^	1900 (745–5723)	<0.001
ALT (IU/L), median (IQR)	35 (20–73)^c^	72 (30–223)	0.008
AST (IU/L), median (IQR)	27 (17–57)^c^	51 (25–117)	0.027
LDH (IU/L), median (IQR)	279 (202–356)^d^	417 (239–698)^i^	0.003
CRP (mg/dL), median (IQR)	0.42 (0.18–1.00)^d^	0.29 (0.13–0.60)^i^	0.153
ESR (mm/h), median (IQR)	12.5 (7.0–33.8)^e^	6.5 (3.3–9.8)^i^	0.002
Ferritin (ng/mL), median (IQR)	226.9 (89.5–696.3)^f^	109.9 (41.2–168.8)^j^	0.007
Treatment at the time of blood collection, no. (%)			0.415
Without treatment	36 (27.9%)	9 (36.0%)	−
Under treatment	93 (72.1%)	16 (64.0%)	−
Physician VAS (0–10)	4.0 (3.0–6.0)	3.5 (1.8–5.5)	0.128

*DM: dermatomyositis; IMNM: immune-mediated necrotizing myopathy; IQR: interquartile range; ILD: interstitial lung disease; RP-ILD: rapidly progressive interstitial lung disease; FVC: forced vital capacity; FEV1: forced expiratory volume in 1s; DLco: diffusing capacity of carbon monoxide; MSA: myositis-specific antibody; MDA5: melanoma differentiation-associated gene 5; ARS: aminoacyl-tRNA synthetase; TIF1-γ: transcriptional intermediary factor 1 γ; NXP-2: nuclear matrix protein 2; SAE: small ubiquitin-like modifier activating enzyme; SRP: signal recognition particle; HMGCR: 3-hydroxy-3-methylglutaryl coenzyme A reductase; CK: creatine kinas; ALT: alanine aminotransferase; AST; aspartate aminotransferase; LDH: lactate dehydrogenase; CRP: C-reactive protein; ESR: erythrocyte sedimentation rate; VAS: visual analog scale. ^*a*^Data available for 53 patients; ^*b*^data available for 46 patients; ^*c*^data available for 127 patients; ^*d*^data available for 128 patients; ^*e*^data available for 124 patients; ^*f*^data available for 109 patients; ^*g*^available for 9 patients; ^*h*^available for 8 patients; ^*i*^available for 24 patients; ^*j*^available for 15 patients.*

### Increased Serum Gal-9 Levels in Patients With IIM, Particularly in Anti-MDA5-Positive Patients With DM

**FIGURE 1:**
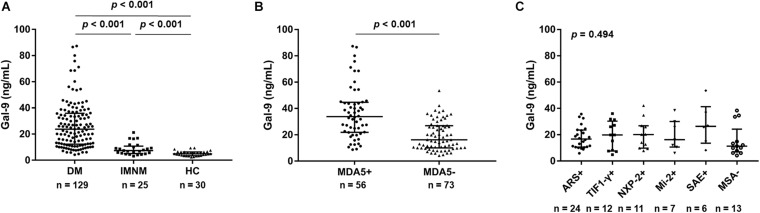
Serum galectin-9 (Gal-9) levels in patients with idiopathic inflammatory myopathies and healthy controls (HCs). **(A)** Patients with dermatomyositis (DM), patients with immune-mediated necrotizing myopathy (IMNM), and HCs. **(B)** Patients with DM divided into anti-melanoma differentiation-associated gene 5 (MDA5)-positive group and anti-MDA5-negative group. **(C)** Anti-MDA5-negative patients stratified by myositis-specific antibodies (MSAs). ARS: aminoacyl-tRN synthetase; TIF1-γ: transcriptional intermediary factor 1 γ; NXP-2: nuclear matrix protein 2; SAE: small ubiquitin-like modifier activating enzyme. Horizontal bars indicate median with interquartile range.

Serum Gal-9 levels were significantly higher in patients with IIM than in HCs [19.8 (10.0–33.6) vs. 4.9 (3.5–6.3) ng/mL, *p* < 0.001]. Additionally, serum Gal-9 levels in patients with DM were more than 3-fold higher than in patients with IMNM [23.7 (12.3–35.9) vs. 7.4 (5.2–10.8) ng/mL, *p* < 0.001] (Figure 1A). Furthermore, after dividing the patients with DM by MSAs, significantly higher serum levels of Gal-9 were observed in the anti-MDA5-positive group than in the anti-MDA5-negative group [33.8 (21.9–44.7) vs. 16.2 (10.0–26.9) ng/mL, *p* < 0.001] (Figure 1B). However, the Kruskal–Wallis H-test (*p* = 0.494) revealed no significant difference between the patients with other MSAs aside from anti-MDA5-antibodies (Figure 1C).

### Significant Association of Serum Gal-9 Levels With RP-ILD and Disease Activity in Anti-MDA5-Positive Patients With DM

**FIGURE 2:**
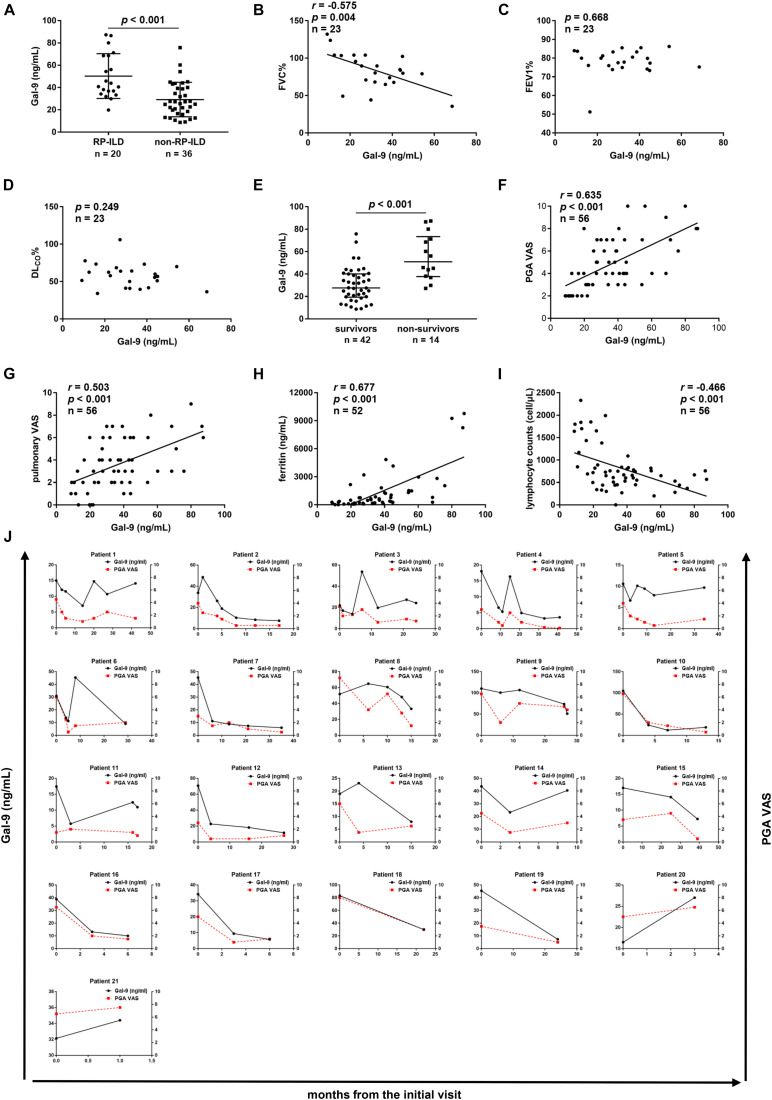
Correlations between serum galectin-9 (Gal-9) levels and clinical features in anti- melanoma differentiation-associated gene 5 (MDA5)-positive patients. **(A)** Serum levels of Gal-9 in patients with rapidly progressive interstitial lung disease (RP-ILD) and patients with non-RP-ILD. **(B–D)** Correlations between serum Gal-9 levels and pulmonary function impairment parameters. **(B)** Serum Gal-9 levels were correlated with forced vital capacity (FVC)% by Pearson’s correlation analysis. **(C)** Serum Gal-9 levels were not correlated with forced expiratory volume in 1s (FEV1)% by Spearman’s correlation analysis. **(D)** Serum Gal-9 levels were not correlated with diffusing capacity of carbon monoxide (DLco)% by Pearson’s correlation analysis. **(E)** Serum levels of Gal-9 in patients who survived and patients who died. **(F,G)** Correlations between serum Gal-9 levels and physician’s global assessment (PGA) visual analog scale (VAS) scores **(F)** and pulmonary VAS scores **(G)** by Spearman’s correlation analysis. **(H)** Serum levels of Gal-9 were correlated with ferritin levels by Spearman’s correlation analysis. **(I)** Serum levels of Gal-9 were correlated with lymphocyte counts by Spearman’s correlation analysis. **(J)** Longitudinal changes in serum levels of Gal-9 and PGA VAS scores over time in 21 anti-MDA5-positive patients. The serum levels of Gal-9 were significantly correlated with PGA VAS scores using the generalized estimating equation model. Horizontal bars indicate median with interquartile range.

We analyzed the association between serum levels of Gal-9 and RP-ILD in anti-MDA5-positive patients with DM. A significant difference was found in serum levels of Gal-9 between patients with RP-ILD (*n* = 20) and patients with non-RP-ILD (*n* = 36) [42.4 (34.9–68.5) vs. 26.4 (17.0–39.8) ng/mL, *p* < 0.001] (Figure 2A). Furthermore, the association between the serum levels of Gal-9 and pulmonary function was analyzed in 23 anti-MDA5-positive DM patients with pulmonary function tests. We observed a negative correlation between serum levels of Gal-9 and FVC% (*r* = −0.575, *p* = 0.004) (Figure 2B). Serum levels of Gal-9 did not correlate with FEV1% (*p* = 0.668) or DL_CO_% (*p* = 0.249) (Figures 2C,D).

To analyze the correlation between serum levels of Gal-9 and the mortality in anti-MDA5-positive DM patients, we compared serum Gal-9 levels between survivors and non-survivors. The follow-up duration ranged from 0.1 to 56 months, and the median follow-up time was 18 months. Forty-two patients survived and 14 patients died. As shown in Figure 2E, a significant decrease was observed in the median serum levels of Gal-9 in patients who survived compared to patients who died [27.6 (19.4–40.1) vs. 51.0 (37.8–73.4) ng/mL, *p* < 0.001].

To determine the relationship between serum Gal-9 levels and disease activity, a cross-sectional study of 56 anti-MDA5-positive patients with DM was conducted. Significant positive correlations were observed between serum Gal-9 levels and PGA VAS scores (*r* = 0.635, *p* < 0.001) (Figure 2F) and pulmonary VAS scores (*r* = 0.503, *p* < 0.001) (Figure 2G). Serum Gal-9 levels were also correlated with muscle VAS scores (*r* = 0.462, *p* < 0.001), cardiac VAS scores (*r* = 0.314, *p* = 0.019), joint VAS scores (*r* = 0.266, *p* = 0.047), and constitutional VAS scores (*r* = 0.380, *p* = 0.004) (Supplementary Figures 1A–D). However, no correlation was observed between the serum levels of Gal-9 and cutaneous VAS scores (*p* = 0.123) or gastrointestinal VAS scores (*p* = 0.084) (Supplementary Figures 1E,F). Furthermore, serum Gal-9 levels were correlated with serum ferritin levels (*r* = 0.677, *p* < 0.001) and lymphocyte counts (*r* = −0.466, *p* < 0.001) in anti-MDA5-positive DM patients (Figures 2H,I).

Additionally, a longitudinal study was conducted in 21 anti-MDA5-positive patients with DM to further explore the association between serum Gal-9 levels and disease activity. We collected 89 serum samples and their corresponding clinical data. The serum levels of Gal-9 were significantly correlated with PGA VAS scores using the generalized estimating equation model (β = 0.041, *p* < 0.001) (Figure 2J).

### PBMCs Isolated From Patients With DM Produced High Levels of Gal-9 and Gal-9 mRNA Levels Correlated With Type-I IFN-Inducible Gene Expression

**FIGURE 3:**
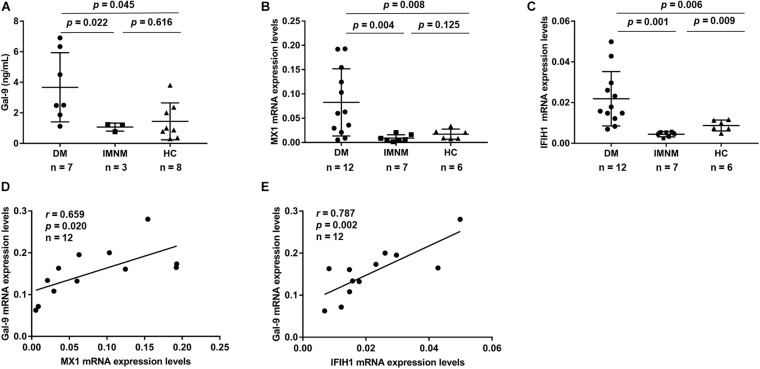
Concentrations of galectin-9 (Gal-9) in peripheral blood mononuclear cells (PBMCs). PBMCs derived from patients with idiopathic inflammatory myopathies and healthy controls (HCs). **(A)** Gal-9 levels in PBMCs derived from patients with dermatomyositis (DM), patients with immune-mediated necrotizing myopathy (IMNM), and HCs. **(B,C)** mRNA expression of type-I interferon-inducible gene myxovirus resistance 1 (MX1) **(B)** and interferon induced with helicase C domain 1 (IFIH1) **(C)** in patients with DM, patients with IMNM, and HCs. **(D,E)** Correlations between Gal-9 and MX1 **(D)** and IFIH1 **(E)** mRNA expression levels in patients with DM. Horizontal bars indicate mean ± standard deviation.

To determine whether PBMCs produce Gal-9 protein, we isolated PBMCs from 7 anti-MDA5-positive patients with DM, 3 patients with IMNM, and 8 HCs. After culturing the PBMCs for 48 h, the levels of Gal-9 in the supernatant were measured by ELISA. As shown in Figure 3A, the levels of Gal-9 in the DM group (3.7 ± 2.3 ng/mL) were higher than those in the IMNM group (1.1 ± 0.3 ng/mL, *p* = 0.022) and HC group (1.4 ± 1.2 ng/mL, *p* = 0.045).

To explore the relation between Gal-9 and type-I IFN, the mRNA levels of Gal-9 and two type-I IFN-inducible genes (MX1 and IFIH1) from PBMCs were measured. PBMCs were isolated from 12 anti-MDA5-positive patients with DM, 7 patients with IMNM, and 6 HCs. The MX1 mRNA levels in patients with DM were higher than those in patients with IMNM (*p* = 0.004) and HCs (*p* = 0.008) (Figure 3B). Similarly, IFIH1 mRNA levels were higher in patients with DM than in patients with IMNM (*p* = 0.001) and HCs (*p* = 0.006) (Figure 3C). Interestingly, Gal-9 mRNA expression was correlated with the mRNA levels of type-I IFN-inducible genes MX1 (*r* = 0.659, *p* = 0.020) and IFIH1 (*r* = 0.787, *p* = 0.002) (Figures 3D,E) in patients with DM.

### Enhanced Gal-9 and Tim-3 Expression in the Lung Tissue of Patients With DM-ILD

**FIGURE 4:**
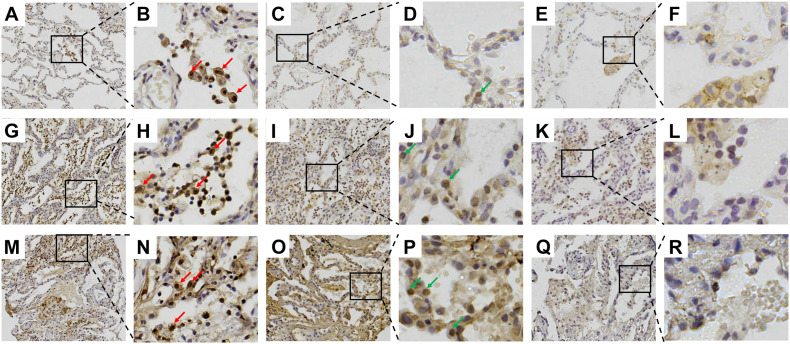
Immunohistochemical staining of galectin-9 (Gal-9) in lung sections. **(A–F)** Surviving 39-year-old female patient with anti-melanoma differentiation-associated gene 5 (MDA5)-positive dermatomyositis (DM) with no interstitial lung disease (ILD). **(G–L)** Surviving 27-year-old male patient with anti-MDA5-positive DM with non-RP-ILD. **(M–R)** Non-surviving 55-year-old male patient with anti-MDA5-positive DM with RP-ILD. **(A,B,G,H,M,N)** Staining with anti-Gal-9 antibody. **(C,D,I,J,O,P)** Staining with anti-T-cell immunoglobulin mucin (Tim)-3 antibody. **(E,F,K,L,Q,R)** Staining with anti-CD44 antibody. The red arrows indicate Gal-9-expressing macrophages. The green arrows indicate Tim-3-expressing alveolar epithelial cells.

Immunohistochemistry revealed that Gal-9 is mainly expressed in macrophages and a small number of alveolar epithelial cells. Furthermore, we counted the Gal-9-positive cells and total cells in five randomly selected visual fields and the expression level of Gal-9 in lung biopsy was defined as the percentage of positive immunostaining cells out of the total number of cells in the tissue area examined at a magnification of 100X. The positive rate of Gal-9 in total cells was about 14% in the patient with no ILD (Figures 4A,B), 27% in the patient with non-RP-ILD (Figures 4G,H) and 38% in the patient with RP-ILD (Figures 4M,N), indicating that Gal-9 expression was more substantial in patients with non-RP-ILD and RP-ILD than those with no ILD. Furthermore, the expression of Tim-3 was elevated in patients with non-RP-ILD (Figures 4I,J) and RP-ILD (Figures 4O,P) compared to those with no ILD (Figures 4C,D), whereas almost no CD44 expression were observed in patients with no ILD (Figures 4E,F), non-RP-ILD (Figures 4K,L), and RP-ILD (Figures 4Q,R).

### Fibroblast Expressed Increased Levels of CCL2 Following Stimulation of Gal-9 *in vitro*

**FIGURE 5:**
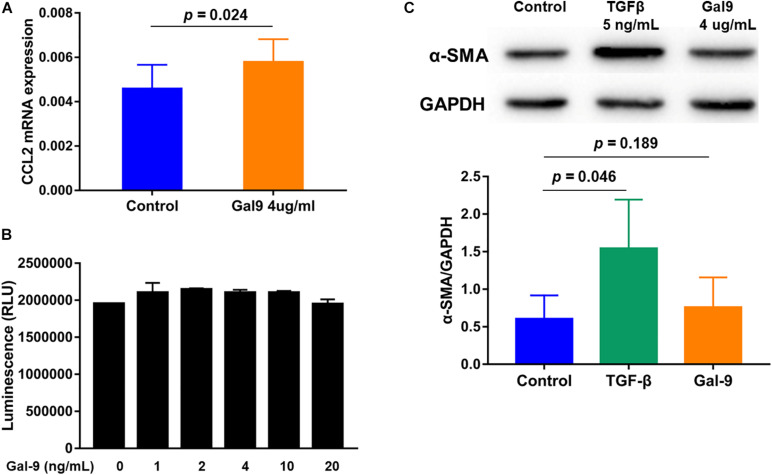
Effects of galectin-9 (Gal-9) on the function of MRC-5 fibroblasts. **(A)** Monocyte chemoattractant protein-1 (CCL2) mRNA levels in Gal-9-treated MRC-5 fibroblasts compared to the control group. **(B)** Proliferation of MRC-5 fibroblasts stimulated with Gal-9 for 24 h. **(C)** α-smooth muscle actin (SMA) protein expression of MRC-5 fibroblasts treated with transforming growth factor-β (TGF-β) or Gal-9. The graphs show the mean ± standard deviation of and each graph determined from at least three independent experiments. GAPDH, glyceraldehyde-3-phosphate dehydrogenase.

To identify the potential roles of Gal-9 in the pathogenesis of DM-ILD, MRC-5 human fibroblasts were stimulated with Gal-9 *in vitro*. We found that MRC-5 fibroblasts stimulated with Gal-9 expressed higher CCL2 (*p* = 0.024) mRNA levels (Figure 5A) compared to control fibroblasts, but no difference was observed in IL-1β, IL-2, IL4, IL-6, IL-8, IL-10, IL-17A, TNF-α, IFN-γ, CCL-18, CXCL4, and CXCL10 (all *p* > 0.05, data not shown). Additionally, the effect of Gal-9 on the proliferation of MRC-5 fibroblasts was explored. Cell viability analysis showed no differences between the control group and Gal-9-treated groups in MRC-5 fibroblasts stimulated for 24 h (all *p* > 0.05, Figure 5B) or 48 h (all *p* > 0.05, data not shown). Fibrosis is caused by the massive deposition of the extracellular matrix. α-SMA protein expression is the primary mediator of fibrosis. TGF-β, a central mediator of fibrosis, has been reported to induce the α-SMA expression. In our study, although TGF-β promoted the expression of α-SMA protein in MRC-5 fibroblasts (*p* = 0.046), Gal-9 did not (*p* = 0.189) (Figure 5C). Overall, these results indicate that MRC-5 fibroblasts expressed increased levels of CCL2 following Gal-9 stimulation. In addition, Gal-9 did not promote proliferation and showed no pro-fibrotic effect on MRC-5 fibroblasts.

## Discussion

This study demonstrated that the serum levels of Gal-9 were significantly increased in patients with IIM, particularly in anti-MDA5-positive patients with DM. Among patients carrying anti-MDA5 antibodies, Gal-9 expression was increased in both the sera and lung tissues of patients with RP-ILD. In addition, a significant correlation was observed between the serum levels of Gal-9 and disease activity in anti-MDA5 positive patients with DM. High serum Gal-9 levels in patients with DM may, at least in part, be derived from PBMCs and were correlated with type-I IFN-inducible gene expression. Furthermore, Gal-9 modulated the production of CCL2 mRNA in MRC-5 fibroblasts *in vitro*.

Elevated serum Gal-9 levels have been detected in various autoimmune disease (Fujita et al., 2020; Matsuoka et al., 2020; Wang et al., 2020). Consistent with the previous studies in patients with juvenile DM (Bellutti Enders et al., 2014; Wienke et al., 2019), our study demonstrated that adult patients with DM exhibited increased serum Gal-9 levels compared to those in HCs. Additionally, the serum levels of Gal-9 were higher in the DM group than in the IMNM group. Serum Gal-9 levels were analyzed by subgrouping patients based on MSAs. Interestingly, anti-MDA5-positive patients with DM exhibited high serum Gal-9 levels.

RP-ILD is a complex and fatal complication in patients with DM, which brings great challenges for clinicians in the clinical management of DM patients. Anti-MDA5 antibodies were demonstrated to be closely linked to RP-ILD (Moghadam-Kia et al., 2016; Chen et al., 2018). It has been reported that 50-100% of DM patients with anti-MDA5 developed ILD, and 39-71% of DM patients with anti-MDA5 developed RP-ILD (Chen et al., 2013; Abe et al., 2017; Moghadam-Kia et al., 2017). Similarly, in our study 89.3% (50/56) of DM patients with anti-MDA5 developed ILD, and 35.7% (20/56) of DM patients with anti-MDA5 developed RP-ILD. Serum levels of Gal-9 in the RP-ILD group were higher than those in the non-RP-ILD group in anti-MDA5-positive patients with DM. We also identified an association between serum Gal-9 levels and pulmonary function impairments in patients with RP-ILD. This suggests that Gal-9 is associated with RP-ILD. Furthermore, in anti-MDA5-positive patients with DM, the serum levels of Gal-9 were linked to disease activity in both cross-sectional and longitudinal studies. Recently, Wienke et al. (2019) demonstrated that serum levels of Gal-9 can be used to distinguish active disease and remission in patients with DM. Thus, the association between serum Gal-9 and clinical features suggests that Gal-9 is an easily detected biomarker for DM disease activity, and possibly also for RP-ILD severity. Furthermore, implementation of serum Gal-9 level analysis into clinical practice may help to evaluate disease activity of anti-MDA5-positive patients with DM.

Gal-9 was shown to be expressed by many immune cells and tissue cells such as monocytes, CD4+ T cells and Treg cells (Wang et al., 2009; Kang et al., 2020; Krautter et al., 2020; Meggyes et al., 2020). We demonstrated that PBMCs isolated from patients with DM produced higher levels of Gal-9 compared to IMNM patients and HCs, suggesting that PBMCs are among the sources of serum Gal-9 in patients with DM. In our study, we use the spontaneous production of Gal-9 to reflect the ability of PBMC to produce Gal-9. Our results may better reflect the *in vivo* situation for the ability of PBMC to produce Gal-9 in DM patients compared to IMNM patients and HCs. IFNs are a large family of cytokines that participate in antiviral response and regulate innate and adaptive immunity, and type-I IFNs were shown to be significantly increased in patients with DM (Lu et al., 2015b). The autoantigen targeted by anti-MDA5 antibody is a type-I IFN-inducible gene. Previous studies have demonstrated an elevated IFN response signature within anti-MDA5 antibody group (Zhang et al., 2019). Similarly, in our study, the expression of type-I IFN-inducible genes MX1 and IFIH1 was enhanced in PBMCs from anti-MDA5-positive patients with DM compared to patients with IMNM and HCs. Additionally, among anti-MDA5-positive patients with DM, we observed positive correlations between Gal-9 mRNA and IFN-inducible genes. Gal-9 was reported to be increased expressed on IFN stimulation (Singh et al., 2007; Park et al., 2011). Furthermore, previous studies showed inhibition of Gal-9 following toll-like receptor (TLR)7- and TLR9-mediated activation of plasmacytoid dendritic cells and B cells in murine lupus models. Gal-9 also inhibited the expression of IFN-α, TNF-α, and IL-6 (Panda et al., 2018). Therefore, Gal-9 may be involved in regulating type-I IFN levels in patients with DM. Although the exact mechanism needs to be further explored, the overactivation of IFN may probably provide biological explanation for the association between Gal-9 and MDA5-positivity.

Furthermore, we investigated Gal-9 expression in the lung tissues of patients with ILD and contribution of Gal-9 to the immunopathogenesis of DM-ILD. Gal-9 expression was upregulated in the lung tissues of RP-ILD. Similarly, a recent study used immunohistochemistry to show that Gal-3 expression is more obvious in patients with DM-ILD than in HCs (Watanabe et al., 2020). Additionally, we confirmed that Tim-3, a Gal-9 ligand, was upregulated in the lung tissues of patients with RP-ILD. A previous study indicated that Tim-3 expression was elevated in patients with idiopathic pulmonary fibrosis (Wang et al., 2019a). These results suggest that the interaction of Gal-9 with Tim-3 may be involved in the pathogenesis of RP-ILD. Particularly, we showed that Gal-9 upregulates CCL2 mRNA levels in MRC-5 fibroblasts *in vitro*. CCL2, a member of the CC chemokine subfamily, was shown to be a potent chemotactic factor for monocytes and macrophages (Behfar et al., 2018). As the major mediator of the initiation and development of inflammatory response, CCL2 was previously associated with pulmonary inflammation (Palchevskiy et al., 2011; Sahin and Wasmuth, 2013). Moreover, previous studies have reported the importance of macrophage activation in the pathophysiology of DM-ILD (Enomoto et al., 2017). Ferritin is a well-known marker of macrophage activation. Consistently, serum Gal-9 levels correlated with serum ferritin levels in anti-MDA5-positive DM patients. Gal-9-stimulated MRC-5 fibroblast expressed an increased levels of inflammatory factor CCL2, whereas the fibrosis marker of MRC-5 fibroblast was unaffected by Gal-9 stimulation *in vitro*. These results suggest a role of Gal-9 in promoting inflammation in DM-ILD, which is consistent with inflammation in the lungs of patients with DM-ILD in the early stage. Therefore, our results suggest that Gal-9 is not only a potential biomarker, but also plays a role in the pathogenesis of DM-ILD.

However, we noticed that other studies indicated the inhibitory effect of Gal-9 in pathological progress of lung disease. For instance, evidence has identified Gal-9 suppressed the growth and induced apoptosis of human lung fibroblast cells in patients with interstitial pneumonia associated with collagen vascular disease and it protected against pulmonary fibrosis of these patients (Matsumoto et al., 2013). Furthermore, Kojima and colleagues reported that Gal-9 attenuated acute lung injury by expanding CD14–CD11b+Gr-1+ plasmacytoid dendritic cell-like macrophages (Kojima et al., 2011). It seems that Gal-9 plays diverse roles in autoimmune diseases. In a study by Zeggar et al. showed that Gal-9 deficiency plays protection roles, and it didn’t alter the TLR7–type I IFN pathway in murine lupus models. Antagonism of Gal-9 is beneficial for the treatment of lupus (Zeggar et al., 2018). However, Panda et al. reported that administration of Gal-9 inhibited splenomegaly in murine lupus models (Panda et al., 2018). The conflicting results might be attributed to different animal models, cell types, and intracellular and extracellular modes action of Gal-9. Further investigation utilizing myositis animal models are needed to confirm the role of Gal-9 in the pathogenesis of DM-ILD.

There were some limitations to this study. First, this was a retrospective study and the number of patients was relatively small, particularly in the follow-up study. Secondly, due to the retrospective design, we are not able to get the data for other core myositis activity measures in these patients. Consequently, the standard core disease activity measures other than VAS score, such as myositis disease activity assessment visual analog scales and manual muscle testing were not evaluated. Thirdly, although PBMCs represent an important source of Gal-9, the specific source cells of Gal-9 were not identified. And DM patients with RP-ILD are usually severe ill, most of them are unable to conduct lung biopsies. Thus, very limited biopsy samples are available. We neither have no enough tissue sample to perform double immunofluorescence staining to precisely determine cell types expressing Gal-9, Tim-3 and CD44. In addition, it has been reported that Gal-9 was increased in the interstitial pneumonia and eosinophilic pneumonia (Asakura et al., 2002; Matsumoto et al., 2013). We speculated that Gal-9 was not specifically up-regulated in DM-ILD, therefore, it is importantly to examined the expression of Gal-9 in other pulmonary disease such as idiopathic pulmonary fibrosis in future investigation.

## Conclusion

In conclusion, we described that serum Gal-9 levels were associated with RP-ILD and disease activity in anti-MDA5-positive patients with DM, and Gal-9 upregulated CCL2 mRNA levels in MRC-5 fibroblasts *in vitro*. Gal-9 may not only represent a new biomarker but also participate in the immunopathogenesis of ILD in patients with DM carrying anti-MDA5 antibodies.

## Data Availability Statement

The raw data supporting the conclusions of this article will be made available by the authors, without undue reservation.

## Ethics Statement

The studies involving human participants were reviewed and approved by the Ethical Review Committee of the China–Japan Friendship Hospital (2019-25-K19). The patients/participants provided their written informed consent to participate in this study.

## Author Contributions

LL contributed to the conceptualization, data curation, formal analysis, investigation, methodology, validation, software, and writing (original draft). Y-MZ contributed to the data curation, methodology, and validation. Y-WS contributed to the data curation and investigation. A-PS and W-LL contributed to the methodology. L-FY contributed to the formal analysis and validation. XL contributed to the methodology and funding acquisition. G-CW contributed to the conceptualization, methodology, funding acquisition, project administration, supervision, and writing (review and editing). Q-LP contributed to the conceptualization, methodology, funding acquisition, project administration, supervision, and writing (review and editing). All authors contributed to the approval of the final manuscript.

## Conflict of Interest

The authors declare that the research was conducted in the absence of any commercial or financial relationships that could be construed as a potential conflict of interest.

## References

[B1] Abe Y. Matsushita M. Tada K. Yamaji K. Takasaki Y. Tamura N. (2017). Clinical characteristics and change in the antibody titres of patients with anti-MDA5 antibody-positive inflammatory myositis. *Rheumatology* 56 1492–1497. 10.1093/rheumatology/kex188 28499006

[B2] Asakura H. Kashio Y. Nakamura K. Seki M. Dai S. Shirato Y. (2002). Selective eosinophil adhesion to fibroblast via IFN-gamma-induced galectin-9. *J. Immunol.* 169 5912–5918. 10.4049/jimmunol.169.10.5912 12421975

[B3] Bacigalupo M. L. Manzi M. Rabinovich G. A. Troncoso M. F. (2013). Hierarchical and selective roles of galectins in hepatocarcinogenesis, liver fibrosis and inflammation of hepatocellular carcinoma. *World J. Gastroenterol.* 19 8831–8849. 10.3748/wjg.v19.i47.8831 24379606 PMC3870534

[B4] Behfar S. Hassanshahi G. Nazari A. Khorramdelazad H. (2018). A brief look at the role of monocyte chemoattractant protein-1 (CCL2) in the pathophysiology of psoriasis. *Cytokine* 110 226–231. 10.1016/j.cyto.2017.12.010 29277337

[B5] Bellutti Enders F. van Wijk F. Scholman R. Hofer M. Prakken B. J. van Royen-Kerkhof A. (2014). Correlation of CXCL10, tumor necrosis factor receptor type II, and galectin 9 with disease activity in juvenile dermatomyositis. *Arthrit. Rheumatol.* 66 2281–2289. 10.1002/art.38676 24756983

[B6] Bi S. Hong P. W. Lee B. Baum L. G. (2011). Galectin-9 binding to cell surface protein disulfide isomerase regulates the redox environment to enhance T-cell migration and HIV entry. *Proc. Natl. Acad. Sci. U S A.* 108 10650–10655. 10.1073/pnas.1017954108 21670307 PMC3127870

[B7] Chen F. Li S. Wang T. Shi J. Wang G. (2018). Clinical Heterogeneity of Interstitial Lung Disease in Polymyositis and Dermatomyositis Patients With or Without Specific Autoantibodies. *Am. J. Med. Sci.* 355 48–53. 10.1016/j.amjms.2017.07.013 29289262

[B8] Chen Z. Cao M. Plana M. N. Liang J. Cai H. Kuwana M. (2013). Utility of anti-melanoma differentiation-associated gene 5 antibody measurement in identifying patients with dermatomyositis and a high risk for developing rapidly progressive interstitial lung disease: a review of the literature and a meta-analysis. *Arthrit. Care Res.* 65 1316–1324. 10.1002/acr.21985 23908005

[B9] Enomoto Y. Suzuki Y. Hozumi H. Mori K. Kono M. Karayama M. (2017). Clinical significance of soluble CD163 in polymyositis-related or dermatomyositis-related interstitial lung disease. *Arthrit. Res. Ther.* 19:9. 10.1186/s13075-016-1214-8 28103926 PMC5248519

[B10] Fujita K. Iwama H. Oura K. Tadokoro T. Samukawa E. Sakamoto T. (2017). Cancer Therapy Due to Apoptosis: Galectin-9. *Int. J. Mol. Sci.* 18:18010074. 10.3390/ijms18010074 28045432 PMC5297709

[B11] Fujita Y. Asano T. Matsuoka N. Temmoku J. Sato S. Matsumoto H. (2020). Differential regulation and correlation between galectin-9 and anti-CCP antibody (ACPA) in rheumatoid arthritis patients. *Arthrit. Res. Ther.* 22:80. 10.1186/s13075-020-02158-3 32293530 PMC7161013

[B12] Golden-Mason L. Rosen H. R. (2017). Galectin-9: Diverse roles in hepatic immune homeostasis and inflammation. *Hepatology* 66 271–279. 10.1002/hep.29106 28195343 PMC5521806

[B13] Gono T. Kawaguchi Y. Satoh T. Kuwana M. Katsumata Y. Takagi K. (2010). Clinical manifestation and prognostic factor in anti-melanoma differentiation-associated gene 5 antibody-associated interstitial lung disease as a complication of dermatomyositis. *Rheumatology* 49 1713–1719. 10.1093/rheumatology/keq149 20498012

[B14] Hoogendijk J. E. Amato A. A. Lecky B. R. Choy E. H. Lundberg I. E. Rose M. R. (2004). 119th ENMC international workshop: trial design in adult idiopathic inflammatory myopathies, with the exception of inclusion body myositis, 10-12 October 2003, Naarden, The Netherlands. *Neuromuscul. Disord.* 14 337–345. 10.1016/j.nmd.2004.02.006 15099594

[B15] Ishikawa Y. Iwata S. Hanami K. Nawata A. Zhang M. Yamagata K. (2018). Relevance of interferon-gamma in pathogenesis of life-threatening rapidly progressive interstitial lung disease in patients with dermatomyositis. *Arthritis Res. Ther.* 20:240. 10.1186/s13075-018-1737-2 30367666 PMC6235206

[B16] Jiao Q. Qian Q. Zhao Z. Fang F. Hu X. An J. (2016). Expression of human T cell immunoglobulin domain and mucin-3 (TIM-3) and TIM-3 ligands in peripheral blood from patients with systemic lupus erythematosus. *Arch. Dermatol. Res.* 308 553–561. 10.1007/s00403-016-1665-4 27394439

[B17] Johnson C. Pinal-Fernandez I. Parikh R. Paik J. Albayda J. Mammen A. (2016). Assessment of Mortality in Autoimmune Myositis With and Without Associated Interstitial Lung Disease. *Lung* 194 733–737. 10.1007/s00408-016-9896-x 27166633 PMC11678787

[B18] Kang J. Wei Z. F. Li M. X. Wang J. H. (2020). Modulatory effect of Tim-3/Galectin-9 axis on T-cell-mediated immunity in pulmonary tuberculosis. *J. Biosci.* 45:60. 32345786

[B19] Katoh S. Ikeda M. Shimizu H. Abe M. Ohue Y. Mouri K. (2015). Increased Galectin-9 Concentration and Number of CD4+Foxp3high+Cells in Bronchoalveolar Lavage Fluid of Patients with Cryptogenic Organizing Pneumonia. *Lung* 193 683–689. 10.1007/s00408-015-9775-x 26249221

[B20] Kojima K. Arikawa T. Saita N. Goto E. Tsumura S. Tanaka R. (2011). Galectin-9 attenuates acute lung injury by expanding CD14- plasmacytoid dendritic cell-like macrophages. *Am. J. Respirat. Crit. Care Med.* 184 328–339. 10.1164/rccm.201010-1566OC 21562126

[B21] Krautter F. Recio C. Hussain M. Lezama D. Maione F. Chimen M. (2020). Characterisation of endogenous Galectin-1 and -9 expression in monocyte and macrophage subsets under resting and inflammatory conditions. *Biomed. Pharmacother.* 130:110595. 10.1016/j.biopha.2020.110595 32771893

[B22] Li S. Ge Y. Yang H. Wang T. Zheng X. Peng Q. (2019). The spectrum and clinical significance of myositis-specific autoantibodies in Chinese patients with idiopathic inflammatory myopathies. *Clin. Rheumatol.* 38 2171–2179. 10.1007/s10067-019-04503-7 30863950

[B23] Lu X. McCoy K. S. Xu J. Hu W. Chen H. Jiang K. (2015a). Galectin-9 ameliorates respiratory syncytial virus-induced pulmonary immunopathology through regulating the balance between Th17 and regulatory T cells. *Virus Res.* 195 162–171. 10.1016/j.virusres.2014.10.011 25451068

[B24] Lu X. Peng Q. Wang G. (2015b). Discovery of new biomarkers of idiopathic inflammatory myopathy. *Clin. Chim. Acta* 444 117–125. 10.1016/j.cca.2015.02.007 25681646

[B25] Lundberg I. E. Tjarnlund A. Bottai M. Werth V. P. Pilkington C. Visser M. (2017). 2017 European League Against Rheumatism/American College of Rheumatology classification criteria for adult and juvenile idiopathic inflammatory myopathies and their major subgroups. *Ann. Rheum. Dis.* 76 1955–1964. 10.1136/annrheumdis-2017-211468 29079590 PMC5736307

[B26] Matsuda S. Kotani T. Ishida T. Fukui K. Fujiki Y. Suzuka T. (2020). Exploration of pathomechanism using comprehensive analysis of serum cytokines in polymyositis/dermatomyositis-interstitial lung disease. *Rheumatology* 59 310–318. 10.1093/rheumatology/kez301 31321420

[B27] Matsumoto N. Katoh S. Yanagi S. Arimura Y. Tokojima M. Ueno M. (2013). A possible role of galectin-9 in the pulmonary fibrosis of patients with interstitial pneumonia. *Lung* 191 191–198. 10.1007/s00408-012-9446-0 23321864

[B28] Matsuoka N. Fujita Y. Temmoku J. Furuya M. Y. Asano T. Sato S. (2020). Galectin-9 as a biomarker for disease activity in systemic lupus erythematosus. *PLoS One* 15:e0227069. 10.1371/journal.pone.0227069 31986153 PMC6984724

[B29] Meggyes M. Szereday L. Bohonyi N. Koppan M. Szegedi S. Marics-Kutas A. (2020). Different Expression Pattern of TIM-3 and Galectin-9 Molecules by Peripheral and Peritoneal Lymphocytes in Women with and without Endometriosis. *Int. J. Mol. Sci.* 21:21072343. 10.3390/ijms21072343 32231038 PMC7177301

[B30] Moghadam-Kia S. Oddis C. V. Sato S. Kuwana M. Aggarwal R. (2016). Anti-Melanoma Differentiation-Associated Gene 5 Is Associated With Rapidly Progressive Lung Disease and Poor Survival in US Patients With Amyopathic and Myopathic Dermatomyositis. *Arthritis Care Res.* 68 689–694. 10.1002/acr.22728 26414240 PMC4864500

[B31] Moghadam-Kia S. Oddis C. Sato S. Kuwana M. Aggarwal R. (2017). Antimelanoma Differentiation-associated Gene 5 Antibody: Expanding the Clinical Spectrum in North American Patients with Dermatomyositis. *J. Rheumatol.* 44 319–325. 10.3899/jrheum.160682 28089977

[B32] Palchevskiy V. Hashemi N. Weigt S. S. Xue Y. Y. Derhovanessian A. Keane M. P. (2011). Immune response CC chemokines CCL2 and CCL5 are associated with pulmonary sarcoidosis. *Fibrogen. Tissue Repair* 4:10. 10.1186/1755-1536-4-10 21463523 PMC3080805

[B33] Panda S. K. Facchinetti V. Voynova E. Hanabuchi S. Karnell J. L. Hanna R. N. (2018). Galectin-9 inhibits TLR7-mediated autoimmunity in murine lupus models. *J. Clin. Invest.* 128 1873–1887. 10.1172/JCI97333 29611821 PMC5919878

[B34] Park W. S. Jung W. K. Park S. K. Heo K. W. Kang M. S. Choi Y. H. (2011). Expression of galectin-9 by IFN-gamma stimulated human nasal polyp fibroblasts through MAPK, PI3K, and JAK/STAT signaling pathways. *Biochem. Biophys. Res. Commun.* 411 259–264. 10.1016/j.bbrc.2011.06.110 21723260

[B35] Sahin H. Wasmuth H. E. (2013). Chemokines in tissue fibrosis. *Biochim. Biophys. Acta Mol. Basis Dis.* 1832 1041–1048. 10.1016/j.bbadis.2012.11.004 23159607

[B36] Selva-O’Callaghan A. Pinal-Fernandez I. Trallero-Araguás E. Milisenda J. Grau-Junyent J. Mammen A. (2018). Classification and management of adult inflammatory myopathies. *Lancet Neurol.* 17 816–828. 10.1016/s1474-4422(18)30254-0 30129477 PMC11646336

[B37] Singh M. K. Scott T. F. LaFramboise W. A. Hu F. Z. Post J. C. Ehrlich G. D. (2007). Gene expression changes in peripheral blood mononuclear cells from multiple sclerosis patients undergoing beta-interferon therapy. *J. Neurol. Sci.* 258 52–59. 10.1016/j.jns.2007.02.034 17467740

[B38] Thiemann S. Baum L. G. (2016). Galectins and Immune Responses-Just How Do They Do Those Things They Do? *Annu. Rev. Immunol.* 34 243–264. 10.1146/annurev-immunol-041015-055402 26907217

[B39] Travis W. D. Costabel U. Hansell D. M. King T. E. Jr. Lynch D. A. Nicholson A. G. (2013). An official American Thoracic Society/European Respiratory Society statement: Update of the international multidisciplinary classification of the idiopathic interstitial pneumonias. *Am. J. Respir. Crit. Care Med.* 188 733–748. 10.1164/rccm.201308-1483ST 24032382 PMC5803655

[B40] van den Hoogen L. L. van Roon J. A. G. Mertens J. S. Wienke J. Lopes A. P. de Jager W. (2018). Galectin-9 is an easy to measure biomarker for the interferon signature in systemic lupus erythematosus and antiphospholipid syndrome. *Ann. Rheum. Dis.* 77 1810–1814. 10.1136/annrheumdis-2018-213497 30185417

[B41] Wang F. Wan L. Zhang C. Zheng X. Li J. Chen Z. K. (2009). Tim-3-Galectin-9 pathway involves the suppression induced by CD4+CD25+ regulatory T cells. *Immunobiology* 214 342–349. 10.1016/j.imbio.2008.10.007 19362679

[B42] Wang K. Zhao J. Chen Z. Li T. Tan X. Zheng Y. (2019b). CD4+CXCR4+ T cells as a novel prognostic biomarker in patients with idiopathic inflammatory myopathy-associated interstitial lung disease. *Rheumatology* 58 511–521. 10.1093/rheumatology/key341 30508148

[B43] Wang Y. Kuai Q. Gao F. Wang Y. He M. Zhou H. (2019a). Overexpression of TIM-3 in Macrophages Aggravates Pathogenesis of Pulmonary Fibrosis in Mice. *Am. J. Respir. Cell Mol. Biol.* 61 727–736. 10.1165/rcmb.2019-0070OC 31162951

[B44] Wang Y. Song L. Sun J. Sui Y. Li D. Li G. (2020). Expression of Galectin-9 and correlation with disease activity and vascular endothelial growth factor in rheumatoid arthritis. *Clin. Exp. Rheumatol.* 38 654–661. 31820713

[B45] Watanabe E. Kato K. Gono T. Chiba E. Terai C. Kotake S. (2020). Serum levels of galectin-3 in idiopathic inflammatory myopathies: a potential biomarker of disease activity. *Rheumatology* 60 322–332. 10.1093/rheumatology/keaa305 32770187

[B46] Wienke J. Bellutti Enders F. Lim J. Mertens J. S. van den Hoogen L. L. Wijngaarde C. A. (2019). Galectin-9 and CXCL10 as Biomarkers for Disease Activity in Juvenile Dermatomyositis: A Longitudinal Cohort Study and Multicohort Validation. *Arthrit. Rheumatol.* 71 1377–1390. 10.1002/art.40881 30861625 PMC6973145

[B47] Wiersma V. R. Clarke A. Pouwels S. D. Perry E. Abdullah T. M. Kelly C. (2019). Galectin-9 Is a Possible Promoter of Immunopathology in Rheumatoid Arthritis by Activation of Peptidyl Arginine Deiminase 4 (PAD-4) in Granulocytes. *Int. J. Mol. Sci.* 20:20164046. 10.3390/ijms20164046 31430907 PMC6721145

[B48] Wiersma V. R. de Bruyn M. Helfrich W. Bremer E. (2013). Therapeutic potential of Galectin-9 in human disease. *Med. Res. Rev.* 33(Suppl. 1), E102–E126. 10.1002/med.20249 21793015

[B49] Wu C. Thalhamer T. Franca R. F. Xiao S. Wang C. Hotta C. (2014). Galectin-9-CD44 interaction enhances stability and function of adaptive regulatory T cells. *Immunity* 41 270–282. 10.1016/j.immuni.2014.06.011 25065622 PMC4219323

[B50] Zeggar S. Watanabe K. S. Teshigawara S. Hiramatsu S. Katsuyama T. Katsuyama E. (2018). Role of Lgals9 Deficiency in Attenuating Nephritis and Arthritis in BALB/c Mice in a Pristane-Induced Lupus Model. *Arthritis Rheumatol.* 70 1089–1101. 10.1002/art.40467 29481735

[B51] Zhang S. H. Zhao Y. Xie Q. B. Jiang Y. Wu Y. K. Yan B. (2019). Aberrant activation of the type I interferon system may contribute to the pathogenesis of anti-melanoma differentiation-associated gene 5 dermatomyositis. *Br. J. Dermatol.* 180 1090–1098. 10.1111/bjd.16917 29947075

[B52] Zhu C. Anderson A. C. Kuchroo V. K. (2011). TIM-3 and its regulatory role in immune responses. *Curr. Top Microbiol. Immunol.* 350 1–15. 10.1007/82_2010_84 20700701

[B53] Zhu C. Anderson A. C. Schubart A. Xiong H. Imitola J. Khoury S. J. (2005). The Tim-3 ligand galectin-9 negatively regulates T helper type 1 immunity. *Nat. Immunol.* 6 1245–1252. 10.1038/ni1271 16286920

[B54] Zuo Y. Ye L. Liu M. Li S. Liu W. Chen F. (2020). Clinical significance of radiological patterns of HRCT and their association with macrophage activation in dermatomyositis. *Rheumatology* 59 2829–2837. 10.1093/rheumatology/keaa034 32065646

