# Supplementary material for: Aberrantly Expressed Galectin-9 Is Involved in the Immunopathogenesis of Anti-MDA5-Positive Dermatomyositis-Associated Interstitial Lung Disease
Source: Front Cell Dev Biol. 2021 Mar 25;9:628128. doi: 10.3389/fcell.2021.628128 (PMC8027128; doi:10.3389/fcell.2021.628128)
Supplement: Supplementary file 1 [file Table_1.docx]

Supplementary Material

## Supplementary Tales

# Supplementary Table 1: Forward and reverse primers of genes

| Gene | Forward | Reverse |
| --- | --- | --- |
| Gal-9 | TCTGGGACTATTCAAGGAGGTC | CCACTGGAGCTGAGAACGG |
| MX1 | GGTGGTCCCCAGTAATGTGG | CGTCAAGATTCCGATGGTCCT |
| IFIH1 | TCACAAGTTGATGGTCCTCAAGT | CTGATGAGTTATTCTCCATGCCC |
| CCL2 | AAGGGCTCGCTCAGCCAGATGC | GGAATCCTGAACCCACTTCTGC |
| IL-1β | AGCTACGAATCTCCGACCAC | CGTTATCCCATGTGTCGAAGAA |
| IL-2 | GTCACAAACAGTGCACCTACT | GTGGCCTTCTTGGGCATGTA |
| IL-4 | CTGGTTGGCTTCCTTCACAG | AGCAGTTCCACAGGCACAAG |
| IL-6 | ACTCACCTCTTCAGAACGAATTG | CCATCTTTGGAAGGTTCAGGTTG |
| IL-8 | ACTGAGAGTGATTGAGAGTGGAC | AACCCTCTGCACCCAGTTTTC |
| IL-10 | CTAACCTCATTCCCCAACCA | CTCAGCCTCCCAAAGTGCT |
| IL-17A | CTCAGGCTTCCTTTGGAGATT | TCCTTTCTGGGTTGTGTGGT |
| TNFα | TCTCCCCTGGAAAGGACAC | AAGAGGCTGAGGAACAAGCA |
| IFNγ | GAGTGTGGAGACCATCAAGGA | GTATTGCTTTGCGTTGGACA |
| CCL18 | GGGGGCTGGTTTCAGAATA | CTCCTTGTCCTCGTCTGCAC |
| CXCL4 | TGAAGAATGGAAGGAAAATTTGC | GCAGCTAGTAGCTAACTCTCCAAAAGT |
| CXCL10 | AACCTCCAGTCTCAGCACCATGAA | AGGTACAGCGTAAGGTTCTAGAGAG |
| GADPH | GTTACACCCCAATCTTCATGTCCAC | GCCCAATACGACCAAATCC |

Supplementary Table 2：Baseline characteristics observed in anti-MDA5-positive DM patients and anti-MDA5-negative DM patients

| Characteristics | Anti-MDA5-positive  DM patients | Anti-MDA5-negative  DM patients | p |
| --- | --- | --- | --- |
|  | DM patients (n = 56) | DM patients (n = 73) |  |
| Gender, no. (%) |  |  | 0.530 |
| Female | 37 (66.1%) | 52 (71.2%) |  |
| Male | 19 (33.9%) | 21 (28.8%) |  |
| Age of onset, median (IQR), years | 49.0 (38.0-57.0) | 50.0 (39.0-58.0) | 0.583 |
| Disease duration, median (IQR), months | 3 (2-9) | 12 (3-36) | < 0.001 |
| Clinical features, no. (%) |  |  |  |
| ILD | 50 (89.3%) | 39 (53.4%) | < 0.001 |
| RP-ILD | 20 (35.7%)  %) | 6 (8.2%) | < 0.001 |
| Muscle weakness | 19 (33.9%) | 36 (49.3%) | 0.080 |
| Myalgia | 14 (25.0%) | 22 (30.1%) | 0.519 |
| Mechanic’s hands | 22 (39.3%) | 15 (20.5%) | 0.020 |
| Raynaud’s phenomenon | 4 (7.1%) | 6 (8.2%) | 1.000 |
| Heliotrope rash | 49 (87.5%) | 46 (63.0%) | 0.002 |
| Gottron’s papules | 42 (75.0%) | 27 (37.0%) | < 0.001 |
| Skin ulceration | 19 (33.9%) | 6 (8.2%) | < 0.001 |
| Calcinosis | 3 (5.4%) | 2 (2.7%) | 0.652 |
| Arthritis/arthralgia | 22 (39.3%) | 13 (17.8%) | 0.007 |
| Dysphagia | 4 (7.1%) | 14 (19.2%) | 0.089 |
| Malignancy | 0 (0%) | 1 (1.4%) | 1.000 |
| Pulmonary function test, median (IQR) |  |  |  |
| FVC %  FEV1 % DLco % | 83.8 (68.0-103.4)^a^ | 87.9 (77.2-107.8)^f^ | 0.162 |
| FEV_1_ % | 79.9 (75.3-83.3)^a^ | 79.3 (76.6-84.7)^f^ | 0.490 |
| DLco % | 57.8 (41.7-68.4)^a^ | 70.6 (58.3-87.4)^a^ | 0.008 |
| Laboratory features |  |  |  |
| CK (IU/L), median (IQR) | 65.0 (31.0-152.0)^b^ | 91.0 (42.5-475.5) | 0.079 |
| ALT (IU/L), median (IQR) | 54 (28-103)^b^ | 27 (17-45) | < 0.001 |
| AST (IU/L), median (IQR) | 41 (24-72)^b^ | 21 (15-48) | < 0.001 |
| LDH (IU/L), median (IQR) | 304 (224-391)^c^ | 251 (186-346) | 0.058 |
| CRP (mg/dL), median (IQR) | 0.52 (0.25-1.46)^c^ | 0.49 (0.16-0.92)^g^ | 0.105 |
| ESR (mm/h), median (IQR) | 25.0 (9.5-54.5)^d^ | 10.0 (5.0-18.0)^h^ | < 0.001 |
| Ferritin (ng/mL), median (IQR) | 495.5 (177.3-1441.0)^e^ | 139.2 (67.4-259.9)^i^ | < 0.001 |
| Lymphocyte counts (cell/μL), median (IQR) | 705 (503-848) | 1310 (880-1970)^h^ | < 0.001 |
| Treatment at the time of blood collection, no. (%)  Without treatment  Under treatment  Physician VAS (0-10) |  |  | 0.747 |
| Without treatment | 16 (28.6%) | 19 (26.0%) |  |
| Under treatment | 40 (71.4%) | 54 (74.0%) |  |
| Physician VAS (0-10) | 4 (3-7) | 3 (2-5) | 0.021 |

DM: dermatomyositis; IMNM: immune-mediated necrotizing myopathy; IQR: interquartile range; ILD: interstitial lung disease; RP-ILD: rapidly progressive interstitial lung disease; FVC: forced vital capacity; FEV1: forced expiratory volume in 1s; DLco: diffusing capacity of carbon monoxide; CK: creatine kinas; ALT: alanine aminotransferase; AST; aspartate aminotransferase; LDH: lactate dehydrogenase; CRP: C-reactive protein; ESR: erythrocyte sedimentation rate; VAS: visual analog scale. ^a^Data available for 23 patients; ^b^data available for 54 patients; ^c^data available for 55 patients; ^d^data available for 53 patients; ^e^data available for 52 patients; ^f^data available for 30 patients; ^g^available for 72 patients; ^h^available for 71 patients; ^i^available for 57 patients.

##
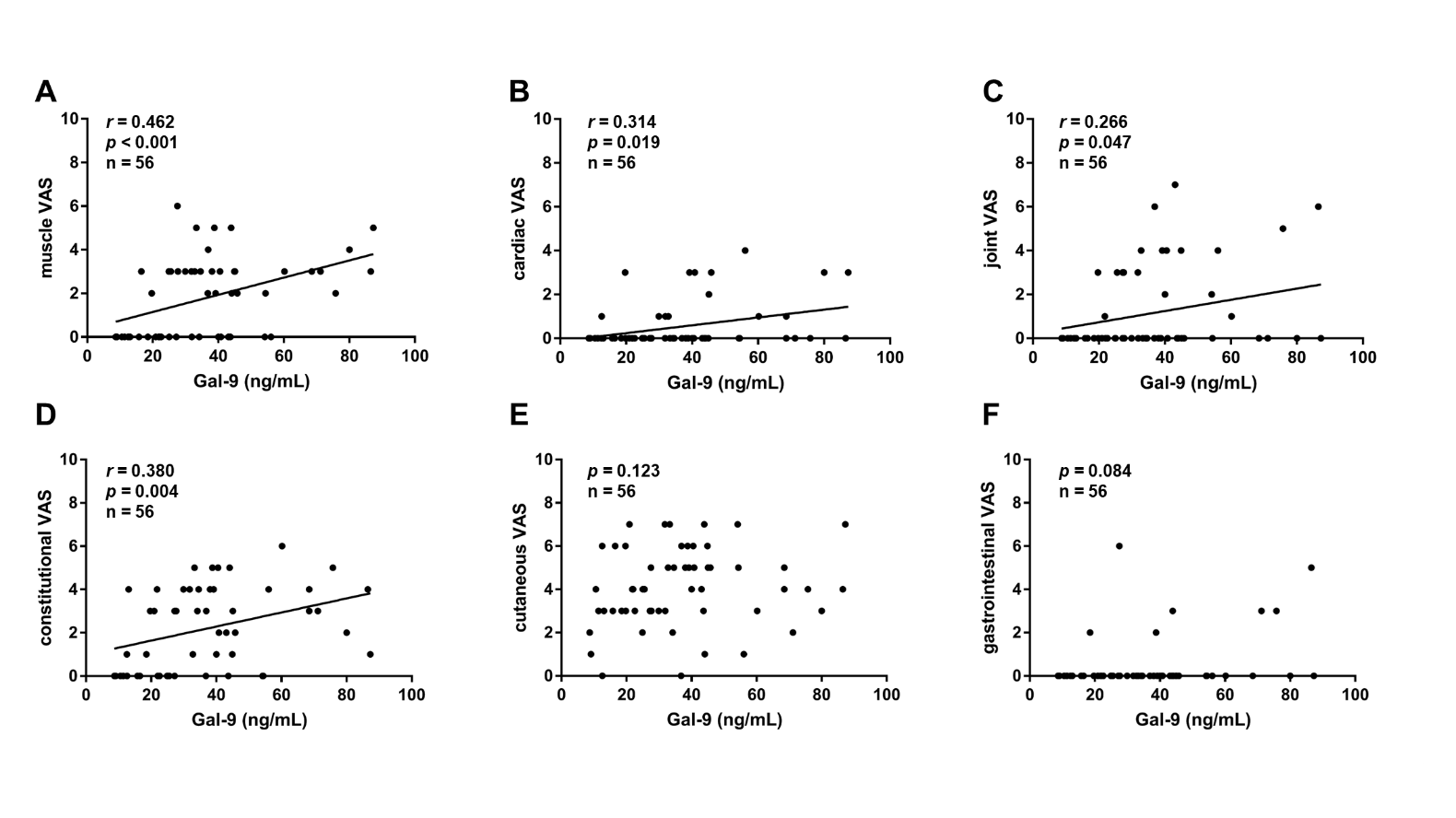
1.2 Supplementary Figures

# Supplementary Figure 1. Correlation between serum Galectin-9 (Gal-9) levels and visual analog scale (VAS) scores. Serum Gal-9 levels were tested in anti-melanoma differentiation-associated gene 5 (MDA5)-positive patients with dermatomyositis. Spearman’s correlation analysis was used for correlation analyses. (A-F) Correlation between serum Gal-9 levels and muscle VAS scores (A), cardiac VAS scores (B), joint VAS scores (C), constitutional VAS scores (D), cutaneous VAS scores (E) and gastrointestinal VAS scores (F).
